# VUScope: a mathematical model for evaluating image-based drug response measurements and predicting long-term incubation outcomes

**DOI:** 10.1093/bioinformatics/btaf679

**Published:** 2026-02-04

**Authors:** Nguyen Khoa Tran, My Ky Huynh, Alexander D Kotman, Martin Jürgens, Thomas Kurz, Sascha Dietrich, Gunnar W Klau, Nan Qin

**Affiliations:** Department of Computer Science, Heinrich Heine University Düsseldorf, Düsseldorf, 40225, Germany; Center for Digital Medicine, Heinrich Heine University Düsseldorf, Düsseldorf, 40599, Germany; Department of Computer Science, Heinrich Heine University Düsseldorf, Düsseldorf, 40225, Germany; Clinic of Hematology, Oncology, and Clinical Immunology, University Hospital of Düsseldorf, Düsseldorf, 40225, Germany; Department of Computer Science, Heinrich Heine University Düsseldorf, Düsseldorf, 40225, Germany; Center for Digital Medicine, Heinrich Heine University Düsseldorf, Düsseldorf, 40599, Germany; Institute of Pharmaceutical and Medicinal Chemistry, Heinrich Heine University Düsseldorf, Düsseldorf, 40225, Germany; Clinic of Hematology, Oncology, and Clinical Immunology, University Hospital of Düsseldorf, Düsseldorf, 40225, Germany; Department of Computer Science, Heinrich Heine University Düsseldorf, Düsseldorf, 40225, Germany; Center for Digital Medicine, Heinrich Heine University Düsseldorf, Düsseldorf, 40599, Germany; Clinic of Hematology, Oncology, and Clinical Immunology, University Hospital of Düsseldorf, Düsseldorf, 40225, Germany; Spatial & Functional Screening Core Facility, University Hospital of Düsseldorf, Düsseldorf, 40225, Germany; Center for Integrated Oncology, Mildred Scheel School of Oncology Aachen-Bonn-Cologne-Düsseldorf, Düsseldorf, 40225, Germany

## Abstract

**Motivation:**

Live-cell imaging-based drug screening increases the likelihood of identifying effective and safe drugs by providing dynamic, high-content, and physiologically relevant data. As a result, it improves the success rate of drug development and facilitates the translation of benchside discoveries to bedside applications. Despite these advantages, no comprehensive metrics currently exist to evaluate dose–time-dependent drug responses. To address this gap, we established a systematic framework to assess drug effects across a range of concentrations and exposure durations simultaneously. This metric enables more accurate evaluation of drug responses measured by live-cell imaging.

**Results:**

We employed treatment concentrations ranging from 0 to 10 μM and performed live-cell imaging-based measurements over a 120-h incubation period. To analyze the experimental data, we developed VUScope, a new mathematical model combining the 4-parameter logistic curve and a logistic function to characterize dose–time-dependent responses. This enabled us to calculate the Growth Rate Inhibition Volume Under the dose–time–response Surface (GRIVUS), which serves as a critical metric for assessing dynamic drug responses. Furthermore, our mathematical model allowed us to predict long-term treatment responses based on short-term drug responses. We validated the predictive capabilities of our model using independent datasets and observed that VUScope enhances prediction accuracy and offers deeper insights into drug effects than previously possible. By integrating VUScope into high-throughput drug screening platforms, we can further improve the efficacy of drug development and treatment selection.

**Availability and implementation:**

We have made VUScope more accessible to users conducting pharmacological studies by uploading a detailed description, example datasets, and the source code to vuscope.albi.hhu.de, https://github.com/AlBi-HHU/VUScope, and https://doi.org/10.5281/zenodo.17610533.

## 1 Introduction

The selection of a 48–72-h incubation period in drug screening experiments is widely adopted as it effectively balances the need for sufficient time for the drug to influence cellular processes with the practicalities of experimental workflows. Specifically, a 72-h incubation period is generally adequate for capturing effects on most mammalian cells, which typically exhibit doubling times in the range of 20–40 h (He *et al.* 2022). Many assays, such as MTT and CellTiter-Glo (CTG), can more reliably detect drug effects after this elapsed time, ensuring high signal-to-noise ratios (Kleijn *et al.* 2016, Abebe *et al.* 2021). Nonetheless, some drugs may act rapidly or exhibit transient effects. An insufficient endpoint might overlook early events or fail to differentiate between immediate and delayed drug responses. Certain compounds, such as epigenetic inhibitors, may require a more extended incubation period to achieve optimal targeted effects (Bauden *et al.* 2015).

To overcome these limitations, employing label-free and noninvasive live-cell imaging techniques facilitates continuous observation of cellular processes over time. This approach enables real-time monitoring of the dynamic effects of drug candidates on living cells, offering insights often missed by traditional endpoint measurement approaches. Ultimately, it provides a more comprehensive understanding of the mechanisms and temporal effects of drugs. However, traditional metrics like IC50, EC50, $E_{\text{max}}$, or area under the dose–response curve (AUC), derived from endpoint assays, do not capture the temporal drug effects observed through live-cell imaging. Consequently, in this study, we aimed to develop a model to calculate dose–time-dependent drug response metrics.

By combining the 4-parameter logistic curve and a logistic function to characterize dose–time-dependent responses, we delivered real-time assessments of drug efficacy and predicted long-term drug responses based on data obtained from shorter incubation periods. Our innovative model, VUScope, has the potential to transform imaging-based drug screening into a cornerstone of pharmacological research.

Hafner *et al.* (2016) introduced the growth rate inhibition (GR) metric to remove the impact of growth rate on drug response. They further extended their dose-dependent GR model by incorporating time as an additional variable, aiming to capture delayed effects, drug adaptation, variable kinetics of drug–target interactions, and drug efflux. However, as discussed in Supplementary Material A, available as supplementary data at *Bioinformatics* online, their GR metric itself is not time-dependent. To address this limitation, we propose a new dose–time-dependent metric derived from VUScope: Growth Rate Inhibition Volume Under the dose–time–response Surface (GRIVUS). To the best of our knowledge, GRIVUS is the first time-dependent drug response metric, enabling the evaluation of dynamic drug responses while reducing reliance on trial-and-error experiments, ultimately optimizing preclinical research. It extends the AUC metric by the additional time dimension and integrates the core idea of the GR metric.

## 2 Materials and methods

This section presents the materials and methods used. Key resources, i.e. experimental models and chemicals, are summarized in Table 1, available as supplementary data at *Bioinformatics* online. Data and code are available on vuscope.albi.hhu.de.

### 2.1 Cell culture

Cells were cultured following Good Cell Culture Practice guidelines. Generally, cells were maintained in Dulbecco’s Modified Eagle’s Medium (DMEM, Gibco, South America) supplemented with 10% Fetal Bovine Serum (FBS, Sigma). The cells were incubated at 37°C in a 5% CO_2_ atmosphere and were passaged as needed when confluency reached approximately 80%. All experiments utilized mycoplasma-free and authenticated cell lines. Authentication was performed regularly using Short Tandem Repeat (STR) analysis by the Genomics & Transcriptomics Laboratory at the Biological and Medical Research Center (BMFZ) at Heinrich Heine University Düsseldorf, Germany.

### 2.2 Imaging

To prevent cell overgrowth at 120h, optimal seeding numbers for each cell line were carefully validated before starting live-cell imaging. Cells were distributed uniformly into 384-well plates using the Multidrop Combi (Thermo Fisher Scientific, Waltham, USA). Cell counts were measured at time zero and again at 3 h, with wells showing major deviations excluded from analysis. Each plate had six healthy control wells to monitor untreated cells. Imaging was carried out every 3 h over 120 h using an Incucyte SX5 (Sartorius, Göttingen, Germany, 10× objective) while maintaining 37°C and 5% CO_2_. Images were analyzed by Incucyte’s Cell-by-Cell module, with resulting counts exported for further analysis and normalized to initial counts of 1 for each cell line–drug pair.

### 2.3 CellTiter-Glo (CTG) luminescent cell viability assay

The CTG reagent (Promega, MA, USA) was prepared following the manufacturer’s instructions for the cell viability assessment. To maintain exponential growth throughout the experiment, the optimal cell concentrations were determined experimentally. Cells were seeded in 384-well plates at a volume of 30 μl per well. After incubation, the CTG reagent was added to lyse the cells, and luminescence was measured using a Spark 10 M microplate reader (Tecan, Männedorf, Switzerland).

### 2.4 Inhibitor libraries and drug screening

Drug screening was conducted at the Spatial & Functional Core Facility of the Medical Faculty at Heinrich Heine University Düsseldorf. Sample preparation and data processing were carried out as previously described (Jeising *et al.* 2024). A library of 18 histone deacetylase inhibitors was used to profile the drug response in four cell lines. All compounds were tested at concentrations ranging from 0 to 10 μM, covering six distinct concentration levels.

### 2.5 Statistical metrics

The mean absolute percentage error (MAPE) is the relative deviation between actual values $A_{t}$ and forecast values $F_{t}$, and is defined as $\text{MAPE}=\frac{100}{n}\sum_{t=1}^{n}\left|\frac{A_{t}-F_{t}}{A_{t}}\right|$.

In contrast to the Pearson correlation coefficient (PCC), the concordance correlation coefficient (CCC) is a measure not only for correlation, but also for agreement and concordance, and is defined as $\text{CCC}=\frac{2\text{PCC}\sigma_{x}\sigma_{y}}{\sigma_{x}^{2}+\sigma_{y}^{2}+{\left(\mu_{x}-\mu_{y}\right)}^{2}}$, where *x* and *y* represent two variables, $\mu_{x}$ and $\mu_{y}$ are their means, and $\sigma_{x}^{2}$ and $\sigma_{y}^{2}$ are their variances. The CCC is commonly reported along with a 95% confidence interval (CI) derived from bootstrap analysis with 100 repetitions.

### 2.6 VUScope: a dose–time–response model

Dose–response relationships are conventionally characterized by the 4-parameter logistic model. To capture time-dependent responses, we incorporate a logistic function to model cell proliferation over time. Our innovative approach combines time dependence with dose dependence, which is mathematically expressed as follows:


$$f(d,t)=\frac{\alpha (t)-\delta (t)}{1+10^{\beta (t)\cdot (d-\gamma (t))}}+\delta (t).$$

Here, $\alpha (t)=\frac{a_{\alpha }\cdot 2^{k_{\alpha }\cdot t}}{(a_{\alpha }-1)+2^{k_{\alpha }\cdot t}}$ with growth rate $k_{\alpha }\in R$ and upper asymptote $a_{\alpha }\in R$ represents the cell count at a time *t* in the absence of drug application. The term $\delta (t)=\frac{a_{\delta }\cdot 2^{k_{\delta }\cdot t}}{(a_{\delta }-1)+2^{k_{\delta }\cdot t}}$ with growth rate $k_{\delta }\in R$ and upper asymptote $a_{\delta }\in R$ represents the cell count at time *t* when subjected to an infinitely high drug dosage. Further, $\beta (t)=k_{\beta }\cdot |\alpha (t)-\delta (t)|$ with scaling factor $k_{\beta }\in R$, $k_{\beta }\geq 0$, reflects the steepness of the curve surrounding γ(t) at time *t*. Finally, $\gamma (t)=k_{\gamma }$ with $k_{\gamma }\in R$ represents the ${\;\text{log}\;}_{10}(\text{IC}50)$ (or ${\;\text{log}\;}_{10}(\text{EC}50)$ for growth-stimulating drugs). The parameters $k_{\alpha },a_{\alpha },k_{\delta },a_{\delta },k_{\beta },k_{\gamma }$ are estimated using the least_squares function in SciPy (Virtanen *et al.* 2020), which we configured to minimize the MAPE. The modeling choices for the time-dependent parameter functions are discussed in Supplementary Material F, available as supplementary data at *Bioinformatics* online.

### 2.7 GRIVUS

After fitting a dose–time–response surface to time-course data, drug response can be quantified with metrics such as the Volume Under the dose–time–response Surface (VUS), a 3D extension of the traditional AUC. Analogous to the GR metric (Hafner *et al.* 2016), we refined the VUS by removing the impact of growth rate to develop the GRIVUS. This refinement involved expressing both the growth rate of the control condition ($k_{\alpha }$) and that of the treated condition ($k_{\delta }$) as a ratio to the control growth rate ($k_{\alpha }$). In cases where $k_{\alpha }<k_{\delta }$, we expressed both values as a ratio to $k_{\delta }$ instead of $k_{\alpha }$, ensuring the exponential prefactors of *t* remain below 1 to prevent numerical instability. This results in $\alpha^{\prime }(t)=\frac{a_{\alpha }\cdot 2^{\frac{k_{\alpha }}{\max\{k_{\alpha },k_{\delta }\}}\cdot t}}{(a_{\alpha }-1)+2^{\frac{k_{\alpha }}{\max\{k_{\alpha },k_{\delta }\}}\cdot t}}$, $\delta^{\prime }(t)=\frac{a_{\delta }\cdot 2^{\frac{k_{\delta }}{\max\{k_{\alpha },k_{\delta }\}}\cdot t}}{(a_{\delta }-1)+2^{\frac{k_{\delta }}{\max\{k_{\alpha },k_{\delta }\}}\cdot t}}$, and $\beta^{\prime }(t)=k_{\beta }\cdot |\alpha^{\prime }(t)-\delta^{\prime }(t)|$ such that GRIVUS is calculated not from the original function f(d,t), but from


$$f^{\prime }(d,t)=\frac{\alpha^{\prime }(t)-\delta^{\prime }(t)}{1+10^{\beta^{\prime }(t)\cdot (d-\gamma (t))}}+\delta^{\prime }(t).$$

**Figure 1.**
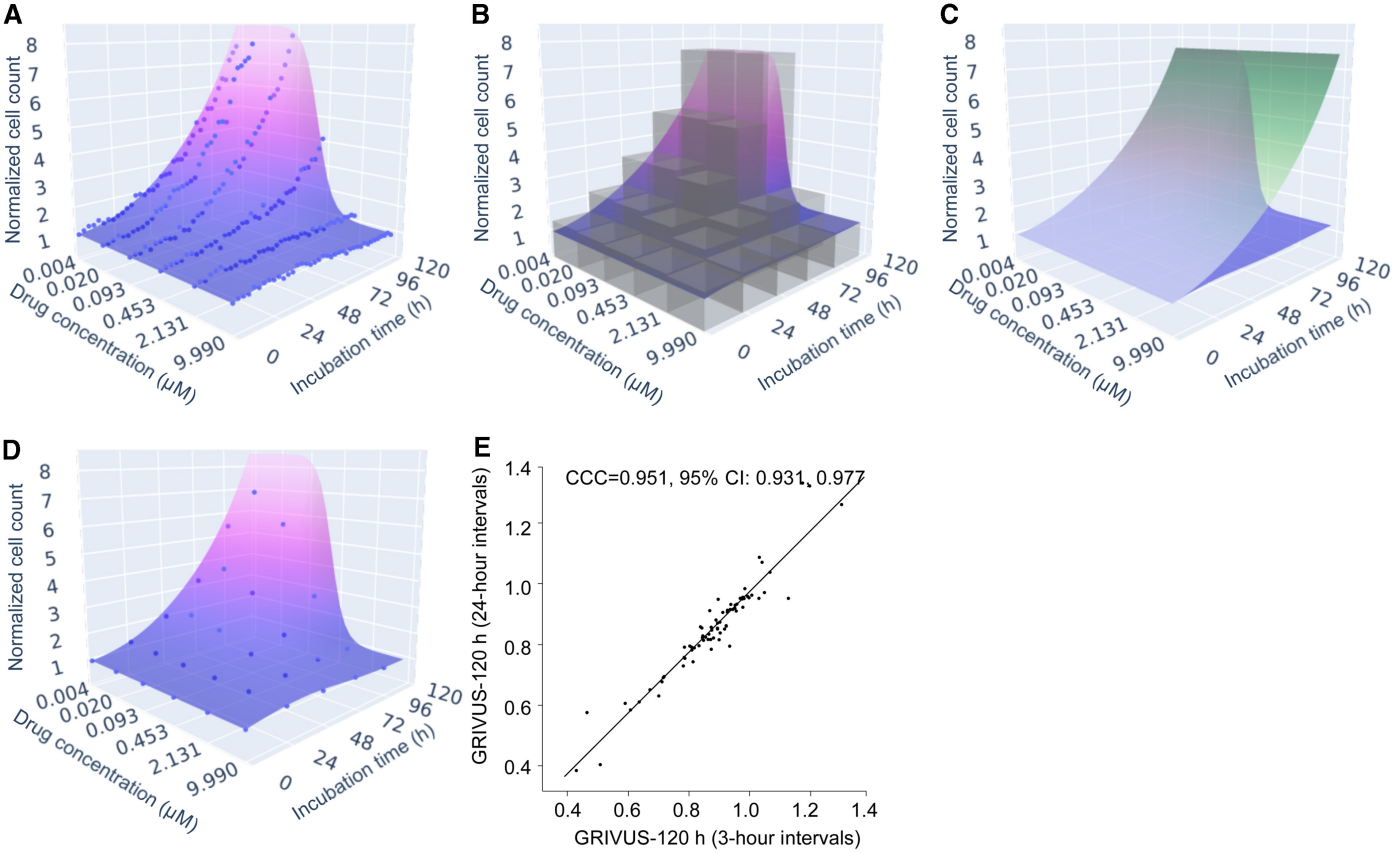
VUScope effectively captures data from imaging-based experiments. (A) 3D fitting of VUScope. The model was constructed using the 4-parameter logistic model in conjunction with a logistic function to represent time-dependent cell growth. (B) The Growth Rate Inhibition Volume Under the dose–time–response Surface (GRIVUS) is calculated using numerical approximation. While the actual number of cuboids is 10 000, this was reduced to 25 for purposes of visualization. Also, we approximate the GRIVUS from above (as seen in the figure) and below, and average both values. (C) The normalized GRIVUS value is calculated by dividing the measured GRIVUS value (blue) by the unaffected GRIVUS value (green). (D) VUScope successfully fits the imaging data using images taken at 24-h intervals. (E) The CCC was computed to compare the GRIVUS values of all cell line–drug pairs calculated from images acquired at 3-h intervals with those from 24-h intervals.

GRIVUS is computed using numerical approximation (Fig. 1B). For data processing, we implemented normalization to minimize bias in the results caused by larger-scale values. Initially, we computed the unaffected GRIVUS value under the assumption that cell proliferation was not influenced by the administered drug at the minimum concentration. We then normalized the GRIVUS value of $f^{\prime }(d,t)$ by dividing it by the unaffected GRIVUS value (Fig. 1C).

## 3 Results

The following section presents results from model fitting as well as extrapolation to predict later time points.

### 3.1 VUScope accurately fits data from live-cell imaging experiments, even with the imaging conducted at 24-h intervals

We used VUScope to accurately fit data from a live-cell imaging experiment, with images acquired every 3 h (Fig. 1A). The resulting 3D-fitted model was quantified by calculating normalized GRIVUS values, as described in Section 2, with calculation strategies illustrated in Fig. 1B and C. To evaluate the accuracy of the VUScope dose–time–response surface, we compared fitted and actual data points using MAPE, a scale-independent metric. Across all cell line–drug pairs, the average MAPE was 8.30%±3.16, highlighting VUScope’s effectiveness for analyzing drug responses from live-cell imaging data. We repeated this analysis with images taken at 24-h intervals (Fig. 1D), observing a slightly higher average MAPE of 8.71%±3.43. Additionally, GRIVUS values for all cell line–drug pairs at both intervals were compared using the CCC. Figure 1E demonstrates nearly perfect concordance, emphasizing the robustness of our fitting model even with reduced data frequency.

### 3.2 VUScope can effectively predict drug effects from short-term treatment data for long-term therapy

**Figure 2.**
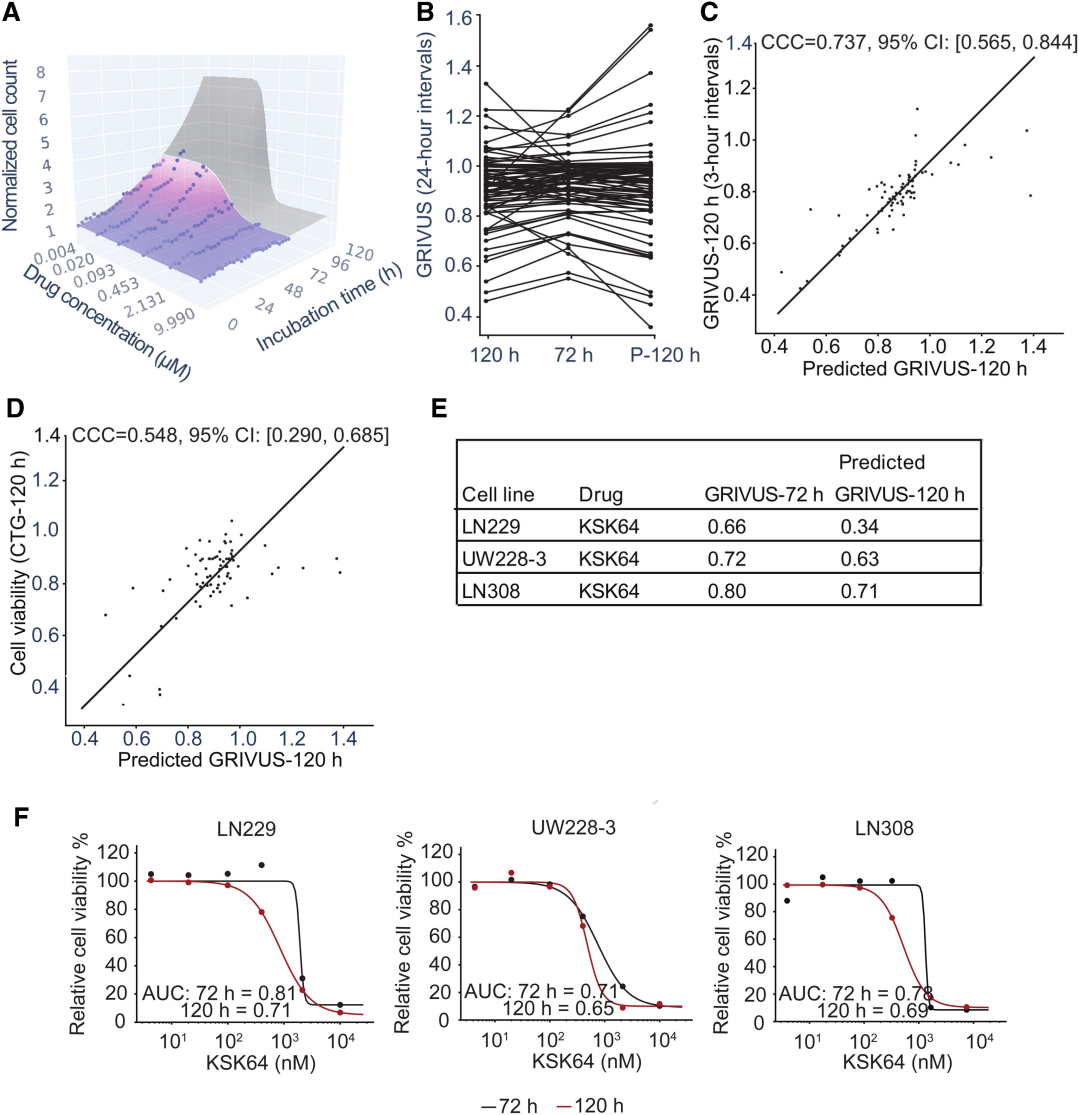
VUScope accurately predicts drug response by analyzing images captured every 3 h. (A) Data from the first 72 h of treatment (blue) were 3D fitted to predict responses after an additional 48 h of incubation (gray). (B) Changes in GRIVUS values observed at 72 h are compared to both the measured and predicted GRIVUS values at 120 h (P-120 h) through paired analysis. (C) Concordance correlation coefficient (CCC) analysis compares the predicted and measured GRIVUS values across all cell line–drug pairs. (D) CCC is also used to compare predicted GRIVUS values at 120 h with cell viability measured by the CTG assay after 120 h of treatment. (E) For KSK64, summarized outcomes are presented using either GRIVUS values at 72 h or predicted GRIVUS at 120 h, demonstrating a larger decline in GRIVUS and suggesting a more specific effect at 120 h. (F) The dose–response curve illustrates the endpoint measurement obtained through the CTG assay after two different treatment durations: 72 h (black) and 120 h (red). The reduction in area under the dose–response curve (AUC) values observed after the 120-h treatment, as compared to the 72-h treatment, underscores the more effective targeted effect of KSK64 following the extended treatment duration.

We evaluated the predictive capability of VUScope by first fitting the dose–time–response model to data collected over the initial 72 h of incubation, using images taken at either 3-h or 24-h intervals. Across all cell line–drug pairs, the average MAPE was 6.14%±2.37 for 3-h intervals and 6.78%±2.79 for 24-h intervals. After calculating the GRIVUS at 72 h, we extracted the optimized parameters and used them to extrapolate the model for predicting GRIVUS at 120 h (Fig. 2A). The model’s 120-h predictions were then assessed against the 120-h fit from the previous subsection. The average MAPE rose only slightly from 8.30%±3.16 to 9.43%±3.80 (3-h intervals) and 9.28%±3.47 (24-h intervals) at 120 h, indicating robust model performance.

In addition, we employed the VUScope fit to the complete 120-h experimental data to calculate the GRIVUS values, which we refer to as *measured* GRIVUS values in the following.

Analysis revealed that, when comparing changes in GRIVUS after 72 h of incubation with the predicted and measured GRIVUS values at 120 h, we observed that the trend, whether there was an increase or decrease in GRIVUS following the 48-h extended incubation, remained consistent across most cell line–drug pairs (Fig. 2B). There was strong concordance between measured and predicted GRIVUS at 120 h as well (Fig. 2C). To independently validate VUScope’s predictive accuracy, we conducted ATP-based cell viability assays at 72 and 120 h of incubation and compared those results with GRIVUS predictions. Despite the methodological differences and resulting variability between ATP-based and live-cell imaging measurements, the observed correlation was strong (Fig. 2D), showing that our model accurately reflects the temporal drug response.

Moreover, we specifically evaluated the targeted effect of KSK64, a newly developed HDAC inhibitor (Friedrich *et al.* 2020), through ATP-based endpoint measurements after either 72 or 120 h of treatment. The resulting dose–response curves corroborated the findings from VUScope (Fig. 2E) and demonstrated an increased targeted effect of KSK64, indicated by lower AUC and GRIVUS values, in three tested cell lines after 120 h of treatment compared to 72 h (Fig. 2F).

Complete results and figures for the data using 24-h intervals are presented in Fig. 1, available as supplementary data at *Bioinformatics* online. Additionally, we investigated VUScope’s ability to predict drug responses over longer incubation times. By analyzing only the first 48 h of data, we predicted responses at 120 h. Despite the challenge of limited data points affecting model fitting, VUScope’s predictions significantly correlated with actual outcomes. Importantly, the predicted changes in drug sensitivity matched the observed directions (Fig. 2, available as supplementary data at *Bioinformatics* online). These results demonstrate that VUScope can accurately forecast long-term drug responses from short-term measurements.

## 4 Discussion

Traditional drug response metrics like IC50, EC50, $E_{\text{max}}$, and AUC capture effects at a single time point, reducing complex temporal drug behaviors to a single value. This simplification often misses nuanced responses such as delayed onset or cumulative effects. These metrics are further influenced by differences between cell types; e.g. fast- and slow-growing cells can respond differently due to their inherent properties rather than the drug itself. Variations in culture conditions and initial seeding density also impact drug sensitivity measurements (Haverty *et al.* 2016).

To address variability due to growth rates and experimental noise, the normalized drug response metric incorporates starting and ending data for treatments (Gupta *et al.* 2020); however, it still overlooks temporal response dynamics. GRIVUS addresses this by combining GR value-based proliferation adjustments with continuous, time-resolved monitoring. This approach enables better characterization of response kinetics, resistance patterns, growth variability, and the distinction between cumulative and transient effects. As a result, using GRIVUS for live-cell or fresh tissue imaging time courses offers deeper insight into dynamic biological processes and enables more comprehensive analyses, often with less sample material (Angeli *et al.* 2024).

Most large-scale studies of cellular response to anti-cancer drugs measure changes in IC50, EC50, $E_{\text{max}}$, or AUC (Barretina *et al.* 2012, Yang *et al.* 2013, Corsello *et al.* 2020). However, when concentration points are insufficient, curve fitting is less reliable, increasing errors in these estimates. Noisy measurements at each dose further increase uncertainty, making Hill slopes and plateaus difficult to estimate and resulting in shifting or imprecise IC50, EC50, $E_{\text{max}}$, and AUC values (Hafner *et al.* 2017). For instance, outliers can greatly influence AUC values (Fig. 3A, available as supplementary data at *Bioinformatics* online), making the 120-h treatment appear more effective than the 72-h one. In contrast, GRIVUS displays high robustness to strong outliers (Fig. 3B, available as supplementary data at *Bioinformatics* online) and still yields accurate predictions (Fig. 3C, available as supplementary data at *Bioinformatics* online).

While prior reports often focus on single-concentration, time-dependent responses (Angeli *et al.* 2024, Colling *et al.* 2024) or on simulated dose–time–response models lacking lab validation (Jackson and Byrne 2000, Murphy *et al.* 2020), our study uniquely integrates substantial experimental (wet lab) data to support model training and utilizes an independent approach to confirm our image-based analyses.

### 4.1 Limitations and future work

Our study deliberately used newly developed HDAC inhibitors, which modify gene expression by altering chromatin structure (Bolden *et al.* 2006). This process may require extended exposure time to accurately assess their targeted effects. Through our dose–time-dependent measurements, incorporating both predicted and measured GRIVUS values, we clearly demonstrated that certain HDAC inhibitors require more than 72 h to achieve their intended effects. This finding indicates that existing studies on HDAC inhibitors that utilize a 72-h treatment duration may underreport their targeted impacts (Ecker *et al.* 2021, Marquardt *et al.* 2023). It also highlights the importance of employing our VUScope and GRIVUS value to determine the appropriate treatment duration rather than relying on a standard 72-h protocol for all cases.

While our model provides valuable insights, it also has limitations that we need to address. Our study focused on a selected range of cell lines and examined the dose–time-dependent drug response to HDAC inhibitors, a specific class of drugs. This focus may influence the robustness and broader applicability of our model. To enhance its effectiveness, we will develop an online tool to assist users in evaluating their live-cell imaging data and optimizing our model simultaneously. By constructing a comprehensive database with adequate training datasets, we can improve the diversity, completeness, and reproducibility of our model.

Currently, our primary focus is on cell count changes, but imaging can yield many additional insights that are worth integrating. Moving forward, it is essential to develop a comprehensive imaging analysis tool that captures data on cell morphology and behavior, growth characteristics in both mono- and co-cultures, and various forms of cell death, such as apoptosis, necrosis, and ferroptosis. Excluding dead cells from cell counts can cause normalized values to drop below 1 and approach 0. In theory, VUScope could already capture this phenomenon, as its growth rates $k_{\alpha }$ and $k_{\delta }$ can take negative values; however, this still needs to be validated with appropriate datasets.

By combining this approach with functional fluorescent reporter assays, we can delve deeper into intracellular organelle dynamics, subcellular protein distribution, activation of signaling pathways, and ion channel activity. This integrated method would lead to a new era of “high-definition” drug response profiling, providing unique insights into therapeutic mechanisms of action and enabling robust quantification of drug responses.

## Supplementary Material

btaf679_Supplementary_Data

## Data Availability

All data related to this manuscript are available and described either within the manuscript or through the URL (vuscope.albi.hhu.de, https://github.com/AlBi-HHU/VUScope, and https://doi.org/10.5281/zenodo.17610533).
