# Supplementary material for: VUScope: a mathematical model for evaluating image-based drug response measurements and predicting long-term incubation outcomes
Source: Bioinformatics. 2026 Feb 4;42(2):btaf679. doi: 10.1093/bioinformatics/btaf679 (PMC12904834; doi:10.1093/bioinformatics/btaf679)
Supplement: btaf679_Supplementary_Data [file btaf679_supplementary_data.pdf]

A GR metrics are not time-dependent

Hafner et al. [6] state that

$$\mathrm{GR}(c,t) = 2^{\left(\frac{\log_2(x(c,t)/x_0)}{\log_2(x_{\mathrm{ctrl}}/x_0)}\right)} - 1 = 2^{\left(1-\frac{S_M\times c^h}{\mathrm{SC}_{50}^h+c^h}-\frac{1}{k}\frac{k_L\times c^h}{\mathrm{LC}_{50}^h+c^h}\right)} - 1,$$

with $x_{\mathrm{ctrl}} = x(0,t)$ and x_0 being the initial cell number, is independent of t. (The meanings of h, S_M, SC_{50}, LC_{50}, and k_L are not relevant in this context.) Hence, $\mathrm{GR}(c,t)$ can be written as $\mathrm{GR}(c)$.
The same goes for the time-dependent GR values Hafner et al. introduce: The authors state that GR values can be evaluated over a time interval $(2\times\Delta t)$ around any time point t based on the equation:

$$\mathrm{GR}(c,t) = 2^{\frac{\log_2(x(c,t+\Delta t)/x(c,t-\Delta t))}{\log_2(x(0,t+\Delta t)/x(0,t-\Delta t))}} - 1$$

However, substituting $x(c,t) = x_0\times\exp\left(t\times k\left(1-\frac{S_M\times c^h}{\mathrm{SC}_{50}^h+c^h}\right) - t\frac{k_L\times c^h}{\mathrm{LC}_{50}^h+c^h}\right)$ yields

$$\begin{aligned}
\mathrm{GR}(c,t) &= 2^{\frac{\log_2\left(\left(x_0\times\exp\left((t+\Delta t)\times k\left(1-\frac{S_M\times c^h}{\mathrm{SC}_{50}^h+c^h}\right)-(t+\Delta t)\frac{k_L\times c^h}{\mathrm{LC}_{50}^h+c^h}\right)\right)/\left(x_0\times\exp\left((t-\Delta t)\times k\left(1-\frac{S_M\times c^h}{\mathrm{SC}_{50}^h+c^h}\right)-(t-\Delta t)\frac{k_L\times c^h}{\mathrm{LC}_{50}^h+c^h}\right)\right)\right)}{\log_2\left(\left(x_0\times\exp\left((t+\Delta t)\times k\left(1-\frac{S_M\times 0^h}{\mathrm{SC}_{50}^h+0^h}\right)-(t+\Delta t)\frac{k_L\times 0^h}{\mathrm{LC}_{50}^h+0^h}\right)\right)/\left(x_0\times\exp\left((t-\Delta t)\times k\left(1-\frac{S_M\times 0^h}{\mathrm{SC}_{50}^h+0^h}\right)-(t-\Delta t)\frac{k_L\times 0^h}{\mathrm{LC}_{50}^h+0^h}\right)\right)\right)}} - 1 \\
&= 2^{\frac{\log_2\left(\left(\exp\left((t+\Delta t)\times k\left(1-\frac{S_M\times c^h}{\mathrm{SC}_{50}^h+c^h}\right)-(t+\Delta t)\frac{k_L\times c^h}{\mathrm{LC}_{50}^h+c^h}\right)\right)/\left(\exp\left((t-\Delta t)\times k\left(1-\frac{S_M\times c^h}{\mathrm{SC}_{50}^h+c^h}\right)-(t-\Delta t)\frac{k_L\times c^h}{\mathrm{LC}_{50}^h+c^h}\right)\right)\right)}{\log_2((\exp((t+\Delta t)\times k))/(\exp((t-\Delta t)\times k)))}} - 1 \\
&= 2^{\frac{\log_2\left(\left(\exp\left((t+\Delta t)\times k\left(1-\frac{S_M\times c^h}{\mathrm{SC}_{50}^h+c^h}\right)-(t+\Delta t)\frac{k_L\times c^h}{\mathrm{LC}_{50}^h+c^h}\right)\right)/\left(\exp\left((t-\Delta t)\times k\left(1-\frac{S_M\times c^h}{\mathrm{SC}_{50}^h+c^h}\right)-(t-\Delta t)\frac{k_L\times c^h}{\mathrm{LC}_{50}^h+c^h}\right)\right)\right)}{\frac{1}{\ln(2)}\times(t+\Delta t)\times k-\frac{1}{\ln(2)}\times(t-\Delta t)\times k}} - 1 \\
&= 2^{\frac{\log_2\left(\left(\exp\left((t+\Delta t)\times k\left(1-\frac{S_M\times c^h}{\mathrm{SC}_{50}^h+c^h}\right)-(t+\Delta t)\frac{k_L\times c^h}{\mathrm{LC}_{50}^h+c^h}\right)\right)/\left(\exp\left((t-\Delta t)\times k\left(1-\frac{S_M\times c^h}{\mathrm{SC}_{50}^h+c^h}\right)-(t-\Delta t)\frac{k_L\times c^h}{\mathrm{LC}_{50}^h+c^h}\right)\right)\right)}{\frac{1}{\ln(2)}\times 2\times\Delta t\times k}} - 1 \\
&= 2^{\frac{\frac{1}{\ln(2)}\times\left((t+\Delta t)\times k\left(1-\frac{S_M\times c^h}{\mathrm{SC}_{50}^h+c^h}\right)-(t+\Delta t)\frac{k_L\times c^h}{\mathrm{LC}_{50}^h+c^h}\right)-\frac{1}{\ln(2)}\times\left((t-\Delta t)\times k\left(1-\frac{S_M\times c^h}{\mathrm{SC}_{50}^h+c^h}\right)-(t-\Delta t)\frac{k_L\times c^h}{\mathrm{LC}_{50}^h+c^h}\right)}{\frac{1}{\ln(2)}\times 2\times\Delta t\times k}} - 1 \\
&= 2^{\frac{(t+\Delta t)\times k\left(1-\frac{S_M\times c^h}{\mathrm{SC}_{50}^h+c^h}\right)-(t+\Delta t)\frac{k_L\times c^h}{\mathrm{LC}_{50}^h+c^h}-(t-\Delta t)\times k\left(1-\frac{S_M\times c^h}{\mathrm{SC}_{50}^h+c^h}\right)-(t-\Delta t)\frac{k_L\times c^h}{\mathrm{LC}_{50}^h+c^h}}{2\times\Delta t\times k}} - 1 \\
&= 2^{\frac{2\times\Delta t\times k\left(1-\frac{S_M\times c^h}{\mathrm{SC}_{50}^h+c^h}\right)-2\times\Delta t\frac{k_L\times c^h}{\mathrm{LC}_{50}^h+c^h}}{2\times\Delta t\times k}} - 1 \\
&= 2^{\frac{k\left(1-\frac{S_M\times c^h}{\mathrm{SC}_{50}^h+c^h}\right)-\frac{k_L\times c^h}{\mathrm{LC}_{50}^h+c^h}}{k}} - 1,
\end{aligned}$$

but this expression is independent of both t and Δt, thus none of the GR metrics $GR(c,t)$ are time-dependent.

B Key resources

Reagent or Resource	Source	Identifier
Experimental Models		
UW228-3	Gift from Dr. Landgraf, University Hospital of Düsseldorf (received: 2015)	RRID: CVCL_0573
LN229	Gift from Dr. Reifenberger, University Hospital of Düsseldorf (received: 2015)	RRID: CVCL_0393
T98G	Gift from Dr. Reifenberger, University Hospital of Düsseldorf (received: 2015)	RRID: CVCL_0556
LN308	Gift from Dr. Reifenberger, University Hospital of Düsseldorf (received: 2015)	RRID: CVCL_0934
All experiments were performed using authenticated cell lines free from mycoplasma contamination. Mycoplasma detection and authentication were performed every six months using the multiplex human cell line authentication test by Multiplexion (Heidelberg, Germany).		
Reagents		
DMEM	Thermo Fisher Scientific	12491023
FBS	Thermo Fisher Scientific	16140071
PBS	Thermo Fisher Scientific	10010023
Dimethylsulfoxid	PanReac AppliChem	A3672,0100
HDAC inhibitors		
Name	Molecular weight (g/mol)	Source
KSK64	406.44	Gift from Dr. Kurz, Heinrich Heine University (received: 2022)
HLK84	333.82	Gift from Dr. Kurz, Heinrich Heine University (received: 2022)
HLK54	301.35	Gift from Dr. Kurz, Heinrich Heine University (received: 2022)
HLK40	412.87	Gift from Dr. Kurz, Heinrich Heine University (received: 2022)
HLK38	397.86	Gift from Dr. Kurz, Heinrich Heine University (received: 2022)
LAK402	494.99	Gift from Dr. Kurz, Heinrich Heine University (received: 2022)
FJKK103	506.96	Gift from Dr. Kurz, Heinrich Heine University (received: 2022)
FJKK133	489.96	Gift from Dr. Kurz, Heinrich Heine University (received: 2022)
SHOK75	421.52	Gift from Dr. Kurz, Heinrich Heine University (received: 2022)
HLK89	504.41	Gift from Dr. Kurz, Heinrich Heine University (received: 2022)
FJKK94	562.04	Gift from Dr. Kurz, Heinrich Heine University (received: 2022)
FJKK81	598.51	Gift from Dr. Kurz, Heinrich Heine University (received: 2022)
MPK169	296.32	Gift from Dr. Kurz, Heinrich Heine University (received: 2022)
YAK376	423.40	Gift from Dr. Kurz, Heinrich Heine University (received: 2022)
YAK169	439.39	Gift from Dr. Kurz, Heinrich Heine University (received: 2022)
FFK24	356.35	Gift from Dr. Kurz, Heinrich Heine University (received: 2022)

Table S1. Summary of experimental models and chemicals.

C Results for predictions with 24-hour interval data

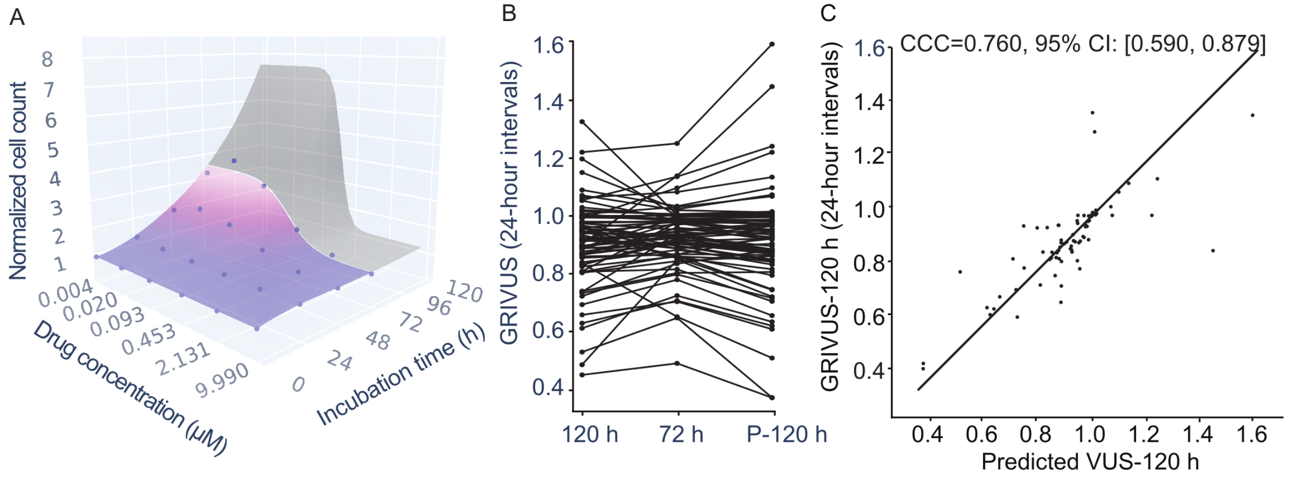

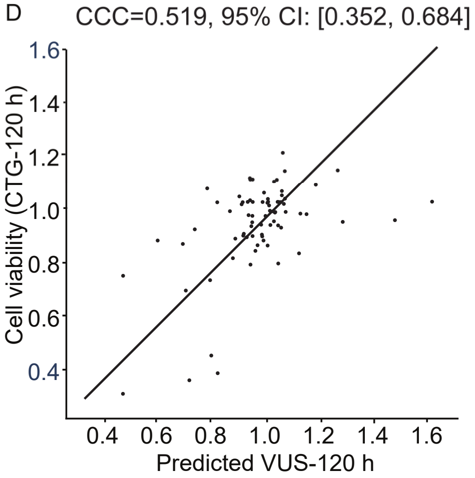

E

Cell line	Drug	GRIVUS-72 h	Predicted GRIVUS-120 h
LN229	KSK64	0.65	0.37
UW228-3	KSK64	0.71	0.62
LN308	KSK64	0.80	0.72

Fig. S1. VUScope accurately predicts drug response by analyzing images captured every 24 hours. (A) Data from the first 72 hours of treatment (blue) were 3D-fitted to predict responses after an additional 48 hours of incubation (gray). (B) Changes in GRIVUS values observed at 72 hours are compared to both the measured and predicted GRIVUS values at 120 hours (P-120 h) through paired analysis. (C) Concordance correlation coefficient (CCC) analysis compares the predicted and measured GRIVUS values across all cell line-drug pairs. (D) CCC is also used to compare predicted GRIVUS values at 120 hours with cell viability measured by the CTG assay after 120 hours of treatment. (E) For KSK64, summarized outcomes are presented using either GRIVUS values at 72 hours or predicted GRIVUS at 120 hours, demonstrating a larger decline in GRIVUS and suggesting a more specific effect at 120 hours.

D Results for predictions with 24-hour interval data

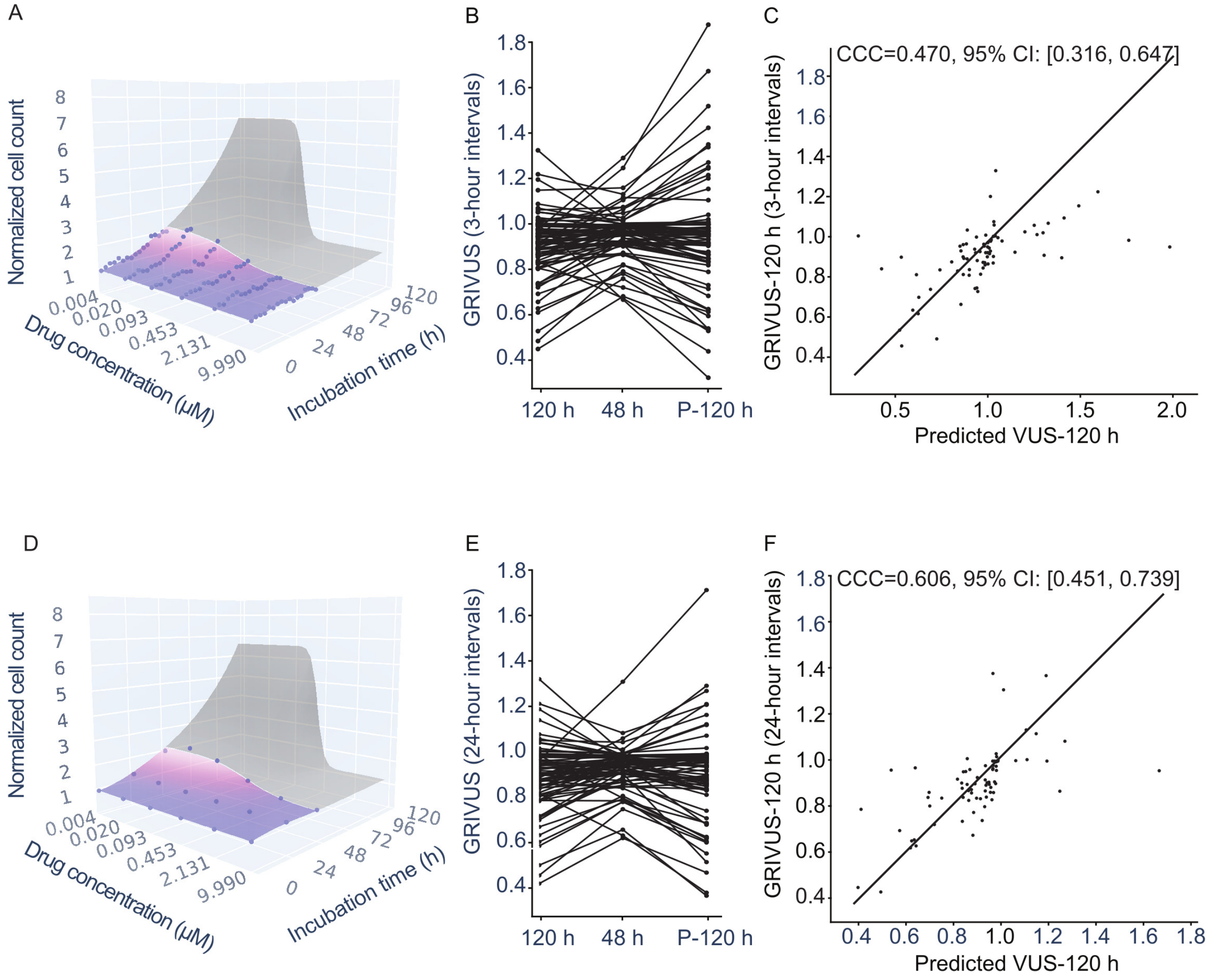

Fig. S2. VUScope predicts drug response by analyzing images during the initial 48 hours of incubation. (A) Images captured at 3-hour intervals in the first 48 hours (blue) were used to build 3D models predicting responses after another 72 hours (gray). (B) Changes in GRIVUS values at 48 hours, calculated from 3-hour interval images, were compared to both measured and predicted GRIVUS values at 120 hours (P-120 h) using paired analysis. (C) Concordance correlation coefficient (CCC) values, based on 3-hour interval images, assess agreement between predicted and measured GRIVUS values across all cell line-drug combinations. (D) Similarly, data from 24-hour interval images in the first 48 hours (blue) predicted response at 120 hours (gray). (E) GRIVUS value changes at 48 hours from 24-hour interval images were compared to measured and predicted values at 120 hours (paired analysis). (F) CCC values from 24-hour interval images compare predicted and measured GRIVUS values for all cell line-drug pairs.

E Outlier susceptibility of IC50, $E_{\mathbf{max}}$, AUC, and GRIVUS

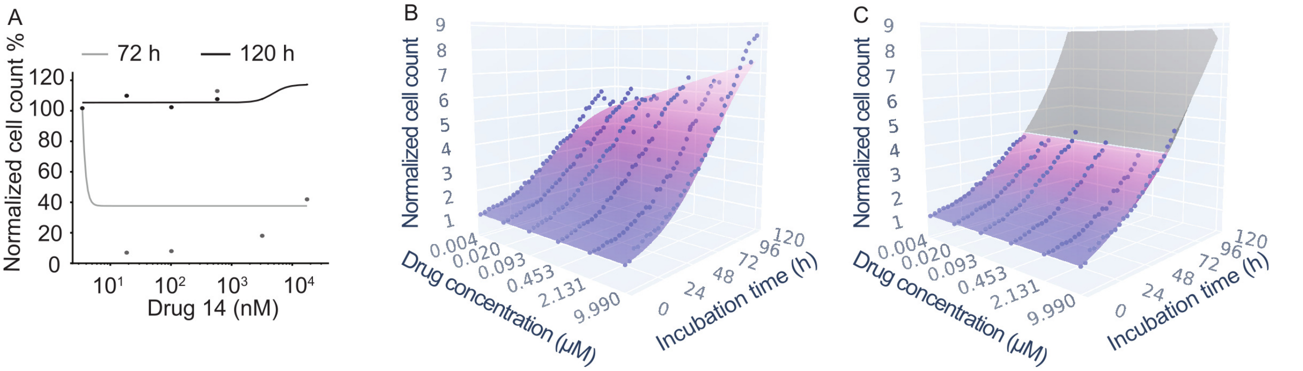

Fig. S3. Outliers can significantly skew IC50, $E_{\max}$, and AUC calculations, while GRIVUS values remain stable. (A) A sample dose-response curve demonstrates that introducing an outlier at 72 hours substantially impairs curve fitting, resulting in unreliable values for IC50, $E_{\max}$, and AUC. This outlier creates a marked difference between 72-hour and 120-hour results: AUC changes from 0.57 to 1.01, IC50 from −2.43 to 0.49, and $E_{\max}$ from 0.43 to −0.10. In contrast, GRIVUS remains robust. Even with several outliers at 120 hours (B), GRIVUS calculations are consistent, with 72-hour (C) and 120-hour values being similar (1.07 and 1.00, respectively).

F Modeling choices

The logistic functions $\alpha(t)$ and $\delta(t)$, used to model cell growth, take the value 1 at $t = 0$ because the normalized cell count is initialized at 1. Setting the asymptote parameter a_α or a_δ to a low value allows a drug with a higher growth rate k_α or k_δ to produce a faster but overall lower effect such that a drug with delayed (lower growth rate) but overall stronger effect (higher asymptotes) can surpass the initially faster, less effective drugs given sufficient time.

In the 4-parameter logistic curve, the maximum slope, given by the derivative at the inflection point $d = \gamma$, is proportional to $|\alpha - \delta|$ with proportionality factor $k_\beta = \frac{\beta \cdot \ln(10)}{4}$. This relationship motivates the choice of $\beta(t)$.

Regarding $\gamma(t)$, it is biologically plausible for the IC50 (or EC50) to change over time because of drug degradation and cellular adaptation mechanisms that commonly develop during extended exposure periods. However, modeling $\gamma(t)$ as a linearly or logistically decreasing function leads to overfitting on the data at hand. A possible reason for the strong performance with a constant $\gamma(t)$ is that at early time points, drug effects are minimal or have not yet manifested across all tested concentrations, meaning that cells initially grow at similar rates regardless of concentration. This results in a flat dose-time-response surface at early time points, i.e., $\alpha(t) - \delta(t) \approx 0$. Consequently, $f(d,t) \approx \delta(t)$, making the model effectively independent of $\gamma(t)$ at early time points. Thus, although $\gamma(t)$ is theoretically expected to be higher at early time points, violating this assumption has little practical impact.
